# Infections with Avian Pathogenic and Fecal *Escherichia coli* Strains Display Similar Lung Histopathology and Macrophage Apoptosis

**DOI:** 10.1371/journal.pone.0041031

**Published:** 2012-07-25

**Authors:** Fabiana Horn, André Mendes Ribeiro Corrêa, Nicolle Lima Barbieri, Susanne Glodde, Karl Dietrich Weyrauch, Bernd Kaspers, David Driemeier, Christa Ewers, Lothar H. Wieler

**Affiliations:** 1 Departamento de Biofísica, Universidade Federal do Rio Grande do Sul, Porto Alegre, Rio Grande do Sul, Brasil; 2 Institute of Microbiology and Epizootics, Freie Universität Berlin, Berlin, Germany; 3 Setor de Patologia Veterinária, Universidade Federal do Rio Grande do Sul, Porto Alegre, Rio Grande do Sul, Brasil; 4 Institut für Veterinär-Anatomie, Freie Universität Berlin, Berlin, Germany; 5 Faculty of Veterinary Medicine, University of Munich, Munich, Germany; 6 Institute of Hygiene and Infectious Diseases of Animals, Justus-Liebig-Universität Giessen, Giessen, Germany; Charité, Campus Benjamin Franklin, Germany

## Abstract

The purpose of this study was to compare histopathological changes in the lungs of chickens infected with avian pathogenic (APEC) and avian fecal (A_fecal_) *Escherichia coli* strains, and to analyze how the interaction of the bacteria with avian macrophages relates to the outcome of the infection. Chickens were infected intratracheally with three APEC strains, MT78, IMT5155, and UEL17, and one non-pathogenic A_fecal_ strain, IMT5104. The pathogenicity of the strains was assessed by isolating bacteria from lungs, kidneys, and spleens at 24 h post-infection (p.i.). Lungs were examined for histopathological changes at 12, 18, and 24 h p.i. Serial lung sections were stained with hematoxylin and eosin (HE), terminal deoxynucleotidyl dUTP nick end labeling (TUNEL) for detection of apoptotic cells, and an anti-O2 antibody for detection of MT78 and IMT5155. UEL17 and IMT5104 did not cause systemic infections and the extents of lung colonization were two orders of magnitude lower than for the septicemic strains MT78 and IMT5155, yet all four strains caused the same extent of inflammation in the lungs. The inflammation was localized; there were some congested areas next to unaffected areas. Only the inflamed regions became labeled with anti-O2 antibody. TUNEL labeling revealed the presence of apoptotic cells at 12 h p.i in the inflamed regions only, and before any necrotic foci could be seen. The TUNEL-positive cells were very likely dying heterophils, as evidenced by the purulent inflammation. Some of the dying cells observed in avian lungs *in situ* may also be macrophages, since all four avian *E. coli* induced caspase 3/7 activation in monolayers of HD11 avian macrophages. In summary, both pathogenic and non-pathogenic fecal strains of avian *E. coli* produce focal infections in the avian lung, and these are accompanied by inflammation and cell death in the infected areas.

## Introduction

Avian pathogenic strains of *Escherichia coli* (APEC) cause several forms of extraintestinal infections in poultry, such as omphalitis in embryos, salpingitis in laying hens, cellulitis (necrotic dermatitis) in broiler chickens, swollen head syndrome, and respiratory tract infections [1]. In any of these examples, infection may become systemic. Respiratory tract infection most likely begins after inhalation of contaminated dust, but only virulent APEC are able to reach the bloodstream and cause generalized infections in otherwise healthy birds.

To cause disease, APEC require adhesins to colonize the lungs and other extraintestinal sites, siderophores to survive within the host fluids, and protectins to evade the host immune system. Knowledge about APEC virulence has grown considerably in the last few years, through the use of experimental infection models [2], the availability of a complete APEC genome [3], the identification of virulence genes [4], [5], [6], [7], [8], [9], [10] and the analysis of their expression [11], [12], [13]. Despite these advances, it is still not possible to predict the virulence of an APEC strain from its genotype [14].

Experimental infection models have been crucial in the study of APEC virulence by allowing investigation of the molecular Koch's postulate that specific inactivation of a gene involved in virulence should lead to a measurable loss in virulence. To test this postulate, null mutants for a potential virulence factor have been compared to wild type bacteria in their ability to colonize chicken organs, such as the lungs, in terms of bacterial counts per gram of tissue [4], [5], [9]. Also using experimental infection models, avian *E. coli* strains from the guts of clinically healthy chickens were found to colonize the avian lung 100–1000 fold less than virulent APEC [14].

Lung histopathology has also been studied in APEC-infected chickens [15], [16]. However, there have previously been no comparisons of the histopathological changes in the lungs of chickens infected with APEC and those infected with non-pathogenic A_fecal_ *E. coli* strains, which have different virulence levels *in vivo*. Here, we used an *in vivo* systemic model in which 5-week-old chickens were infected intratracheally with three different APEC strains and one non-pathogenic A_fecal_ strain in order to analyze lung histopathology. We looked for differences that could account for the ability of a strain to become systemic, by analyzing lung sections for the presence of TUNEL-positive (i.e. dying) cells, in addition to standard HE staining. We also compared the ability of the APEC and A_fecal_ strains to associate with and induce apoptosis in monolayers of HD11 avian macrophages.

## Materials and Methods

### Ethics statement

All animal experiments were approved by the “Landesamt fuer Gesundheit und Soziales” LAGeSo) (G 0220/06) and chickens were killed according to animal welfare norms (Reg. 0220/06).

### Bacterial strains and growth conditions

Three avian *E. coli* strains implicated in colibacillosis and one *E. coli* strain isolated from the microbiota of a healthy chicken were used in this study. MT78 (O2:K1:H5; multilocus sequence type ST95) was isolated in France from the trachea of a chicken with a respiratory tract infection [17]. IMT5155 (O2:K1:H5; ST140, ST complex 95) was isolated in Germany from a septicemic laying hen; it caused systemic infection when inoculated intratracheally in 5-week-old chickens [5]. UEL17 (Ont:H5; ST117) was recovered from the trachea of a septicemic chicken in Brazil [18]. Strain IMT5104 (O8:NM; ST366) was isolated from the intestinal microbiota of a healthy chicken in Germany [14]; IMT5104 genotype revealed an almost absence of virulence factors associated with APEC (Table 1), and it was unable to cause a generalized infection in experimentally infected birds [5]. For being a non-pathogenic A_fecal_ strain, we used IMT5104 as a negative control. These strains were chosen because they associate strongly (MT78 and UEL17) or poorly (IMT5155 and IMT5104) with HD11 avian macrophages (see below). The virulence-associated genes in each strain [2], [14], [19] are shown in Table 1.

**Table 1:** Virulence-associated gene fingerprint of *E. coli* strains.

	*Virulence genes and description*	*Strains*			
		MT78^a^	IMT5155^b^	UEL17^a^	IMT5104^c^
**Adhesins**					
***aatA***	APEC autotransporter toxin	**−**	**+**	**+**	**−**
***afa/draB***	Afimbrial/Dr antigen-specific adhesin	**−**	**−**	**−**	**−**
***csgA***	Cryptic curlin subunit gene	**+**	**+**	**+**	**+**
***ea/I***	ExPEC adhesin I	**+**	**+**	**−**	**−**
***fimC***	Type 1 fimbriae	**+**	**+**	**+**	**−**
***hra***	Heat-resistant agglutinin	**+**	**−**	**+**	**−**
***iha***	Iron-regulated-gene-homologue adhesin	**−**	**−**	**−**	**−**
***papC***	Pilus associated with pyelonephritis	**−**	**−**	**−**	**−**
***sfa/focCD***	S/F1C fimbriae	**−**	**−**	**−**	**−**
***tsh***	Temperature sensitive hemagglutinin	**−**	**+**	**−**	**−**
***mat***	Meningitis associated and temperature regulated fimbriae	**+**	**+**	**+**	**+**
**Invasins**					
***gimB***	Genetic island associated with newborn meningitis	**+**	**+**	**−**	**−**
***ibeA***	Invasion of brain endothelium	**+**	**+**	**−**	**−**
***tia***	Toxigenic invasion locus in ETEC	**−**	**−**	**−**	**−**
**Iron acquisition**					
***chuA***	Heme receptor (*E. coli* haem utilization)	**+**	**+**	**+**	**−**
***fyuA***	Ferric yersinia uptake (yersiniabactin receptor)	**+**	**+**	**−**	**−**
***ireA***	Iron-responsive element	**+**	**+**	**+**	**−**
***iroN***	Catecholate siderophore (salmochelin)	**+**	**+**	**+**	**−**
***irp2***	Iron repressible protein (yersiniabactin synthesis)	**+**	**+**	**−**	**−**
***iucD***	Aerobactin synthesis	**+**	**+**	**+**	**−**
***sitA***	Salmonella iron transport system	**+**	**+**	**+**	**−**
**Protectins/Serum resistance**					
***iss***	Increased serum survival	**+**	**+**	**+**	**−**
***neuC***	K1 capsular polysaccharide biosynthesis	**+**	**+**	**−**	**−**
***kpsMT II***	Group II capsule antigens	**+**	**+**	**−**	**−**
***ompA***	Outer membrane protein A	**+**	**+**	**+**	**+**
***ompT***	Outer membrane protein T	**+**	**+**	**+**	**+**
***traT***	Transfer Protein	**+**	**+**	**+**	**−**
**Toxins**					
***astA***	EAST1 (heat stable cytotoxin associated with enteroaggregative *E. coli*)	**−**	**−**	**−**	**−**
***cnf1/2***	Cytotoxic necrotising factor	**−**	**−**	**−**	**−**
***sat***	Secreted autotransporter toxin	**−**	**−**	**−**	**−**
***vat***	Vacuolating autotransporter toxin	**−**	**+**	**+**	**−**
***hlyA***	Hemolysin A	**−**	**−**	**−**	**−**
***hlyF***	Hemolysin F	**+**	**+**	**+**	**+**
**Others**					
***cvaB/cvaC***	ColV plasmid operon	**+**	**+**	**+**	**−**
***pic***	Serin protease autotransporter	**−**	**−**	**+**	**−**
***Rpai***	Pathogenicity-associated island marker CFTO73	**+**	**+**	**−**	**−**

Positive (+) and negative signs (−) indicate the presence and absence, respectively, of the indicated genes.

a [19];

b [2];

c [14].

Microorganisms were stored in Luria Bertani (LB) broth containing 20% glycerol at −70°C. For experimental procedures, strains were grown in LB broth as described [19]. Bacteria were resuspended in phosphate-buffered saline (PBS) for the animal experiments, or in RPMI medium for the *in vitro* experiments.

Either heat or UV inactivation was used to inactivate bacteria. For heat inactivation LB cultures were heated at 60°C for 30 min, after which the bacteria were harvested as described above. For UV inactivation, bacteria were harvested from LB culture, washed twice in cold PBS, resuspended in cold PBS, and UV irradiated (Bio-Link BLX-254) for 15 min prior to infection.

### Infection of chickens

The virulence of APEC strains MT78 and UEL17 was tested in a systemic chicken infection model, as described by Antão et al. [2]. Five-week-old White Leghorn specific pathogen-free chickens were obtained from Lohmann Selected Leghorn (Lohmann Tierzucht GmbH, Cuxhaven, Germany); chickens were free of distinct pathogens, including Infectious Bronchitis Virus. Briefly, groups of 5-week-old chickens were inoculated intratracheally with 0.5 mL of a PBS suspension containing 10^9^ colony forming units (CFU). Twenty four hours p.i., chickens were euthanized and bacteria were isolated from lungs, kidneys and spleens. The organs were homogenized in sterile PBS and the homogenates plated onto LB agar for colony counting.

To analyze histological sections of lungs, 5-week-old chickens were infected intratracheally with 10^9^ CFU of strains MT78, IMT5155, UEL17 (APEC) or IMT5104 (A_fecal_), or mock-infected with PBS. Twelve, 18 and 24 hours p.i., the animals were euthanized and their lungs dissected, fixed in 8% formaldehyde in PBS for 2–3 days and paraffin-embedded.

### Histological analyses

Sections (4–6 µm) were fixed on slides, deparaffinized, and stained with HE, or labeled by TUNEL or with anti-O2 antibody (for MT78 and IMT5155). Sections were deparaffinized in xylol, and then rehydrated in ethanol (100%, 96%, 80%, and 70%), followed by 2 min in distilled water for HE staining, or Tris-buffered saline (TBS, Tris-HCl 50 mM pH 7.6, NaCl 0.9%) for TUNEL and anti-O2 labeling.

TUNEL labeling was performed as described by Gavrieli et al. [20]. Briefly, deparaffinized sections were incubated with 5–15 µg proteinase K (in TBS) for 15 min at room temperature (RT), washed 4 times with TBS, and then incubated in 2% H_2_O_2_ (in TBS) for 5 min at RT to inactivate endogenous peroxidase. After 3×2 min washes in TBS, the sections were incubated in TBS containing 20% fetal calf serum, 1% BSA and 0.1% gelatin for 30 min at RT. They were then rinsed in TBS for 5 min, incubated in TdT buffer (Tris-HCl 30 mM pH 7.2, sodium cacodylate 140 mM, cobalt chloride 1 mM) for 15 min at RT in a humid chamber, and incubated with terminal desoxynucleotidyl transferase (100 U/mL; Sigma-Aldrich, Germany) and biotinylated dUTP (10 µM; Roche, Mannheim, Germany) in TdT buffer for 60 min at 37°C in a humid chamber. The dUTP labeling reaction was terminated by immersing the slides in TB buffer (sodium chloride 300 mM, sodium citrate 30 mM) twice for 5 min at RT. After rinsing in TBS, samples were covered with streptavidin/biotinylated peroxidase (Dako, Denmark) diluted in TBS according to the manufacturer's instructions and incubated for 30 min at RT. After rinsing twice in TBS, sections were stained with diaminobenzidine-tetrahydrochloride (Sigma-Aldrich, Germany; 0.2 mg/mL DAB in TBS containing 0.005% H_2_0_2_) for 5–10 min at RT. They were dehydrated in ethanol (70%, 80%, 96% and 100%), and xylol and then mounted in Entellan® (Merck).

Rabbit anti-O2 antibody was kindly donated by Dr. Lothar Beutin (National Reference Laboratories for *Escherichia coli*, Federal Institute for Risk Assessment (BfR), Berlin, Germany). Sections were incubated with anti-O2 antibody (diluted 1∶50 in TBS) for 3 h at 37°C in a humid chamber, followed by incubation with goat anti-rabbit serum conjugated to horseradish peroxidase (Dako, Denmark). After washing, they were stained with diaminobenzidine-tetrahydrochloride (Dako, Denmark; 0.5 mg/mL DAB in TBS containing 0.75% H_2_O_2_) for 5–10 min at RT. Finally the sections were counterstained with hematoxylin, dehydrated and mounted in Entellan® (Merck).

### The HD11 chicken macrophage cell line, and infection assays

To analyze APEC-macrophage interactions, we used the chicken macrophage cell line HD11, an avian myelocytomatosis virus (MC29)-transformed chicken macrophage-like cell line [21] that has been well characterized [22], [23], [24] and is believed to be an accurate representation of primary avian macrophages. Macrophages were cultivated at 37°C in 5% CO_2_ in cell culture medium, which consisted of RPMI 1640 medium (Gibco, Grand Island, NY) containing 10% fetal calf serum (FCS, Gibco, Grand Island, NY) and 2 mM glutamine.

For infection assays, macrophages were plated 18 h before infection at 7.5×10^4^ cell/cm^2^ in infection medium, which consisted of RPMI 1640 medium containing 2% fetal calf serum (FCS, Gibco, Grand Island, NY) and 2 mM glutamine. Immediately before infection, cultures were washed once with PBS to remove dead cells, returned to the infection medium, and infected with the APEC strains at multiplicities of infections (MOI) of 150 CFU per cell. After 1 h of incubation at 37°C, the medium was removed, and the cells were washed 3 times with PBS and incubated in infection medium supplemented with 50 µg/ml gentamicin for different lengths of time.

To observe the association between bacteria and macrophages, macrophages were plated on glass coverslips in 24-well plates (2×10^5^ cells per well) and infected with bacteria at a MOI of 150 CFU per cell as described above. After 1 h, the cells were washed and incubated in medium containing gentamicin (50 µg/mL) until 6 h p.i. At this time they were fixed *in situ* with 3.7% formaldehyde in PBS for 10 min at RT and stained with Giemsa (12 drops of Giemsa's solution (Merck, Germany) in 10 mL distilled water) for 20 min at RT. Samples were then washed with water and observed under a light microscope at ×1,000.

For TUNEL staining of APEC-infected HD11 cells, macrophages were infected, fixed at 8 h p.i. with 3.7% formaldehyde in PBS and stained using an *in situ* Cell Death Detection Kit (Roche, Germany). Samples were observed under a Leica fluorescence microscope using the Y3-filter.

### Analyses of caspase activation

To investigate if infection of HD11 macrophages by IMT5155, MT78, UEL17 and IMT5104 activated apoptosis, HD11 macrophages were plated in 6-well plates (7×10^5^ cells per well) and infected at an MOI of 150 CFU per cell as described above (Section 2.5). At 6 h p.i., cell extracts were prepared and assayed for caspase-3/7 activation as described [19]. As a positive control, macrophages were irradiated with 0.02 joules of UV light (Bio-Link BLX-254) and then incubated in infection medium for 6 h.

In the experiments with polymixin B, cells received 5 µg/mL of polymixin B (from a 5 mg/mL stock solution) at the time of infection, and the drug was replaced after the cells were washed.

## Results

### Virulence of MT78 and UEL17 in a systemic chicken model

To analyze the ability of the MT78 and UEL17 strains to cause systemic infection, five chickens were inoculated intratracheally with 10^9^ CFU of each strain. Twenty-four hours p.i., the animals were euthanized, and several organs were analyzed for lesions and bacteria were isolated from lungs, spleens and kidneys. Table 2 shows the mean lesion scores in air sacs, lungs, hearts, livers and spleens. Strain MT78 colonized the lungs and spread to the kidneys and spleen in all animals (Table 3); the CFU counts for MT78 were similar to those observed for IMT5155 [2], [14]. In contrast, colonization by strain UEL17 was restricted to the lungs (Table 2), as was the case for the non-pathogenic strain IMT5104 [5]. The inability of UEL17 to reach internal organs following intratracheal infection was confirmed in another experiment with 10 birds (data not shown).

**Table 2:** Mean organ lesion scores in chickens infected with APEC MT78 and UEL17.

	*Mean lesion scores*					
Strain^a^	Air sacs^b^	Lungs^b^	Heart^b^	Liver^b^	Spleen^b^	All organs^b^
MT78	2.025±0.427	2.5±0.884	1.05±1.168	0±0	1±0	6.575±2.157
UEL17	0.975±0.503	3.25±1.027	0±0	0±0	1±0	5.225±1.393

a *n* = 5 chickens per strain at 24 h p.i.

b Organ lesions were scored as described [2]: air sacs 0–3; lungs 0–5; heart 0–3; liver 0–2 and spleen 0–1; maximum additive lesion score: 14.

**Table 3:** Ability of APEC MT78 and UEL17 to colonize lungs, spleen and kidneys of chickens inoculated via trachea.

Strain	*n*	Lungs*	*n*	Spleen*	*n*	Kidneys*
MT78	5/5	1.03×10^8^±9.07×10^7^	5/5	7.31×10^5^±6.18×10^5^	5/5	2.51×10^6^±4.25×10^6^
UEL17	4/5	2.74×10^6^±3.04×10^6^	0/5	0±0	2/5	2.1×10^4^±4.20×10^4^

Five chickens were inoculated per strain in one experimental infection.

*n* = number of animals from which *E. coli* was recovered from the specified organ.

* mean number of bacteria (CFU) recovered per g tissue.

### Histological analysis of lung tissue sections from APEC-infected chickens

We performed histological analyses of the chicken lungs 12 h, 18 h or 24 h after intratracheal infection with APEC strains MT78, UEL17 and IMT5155 and non-pathogenic A_fecal_ strain IMT5104, and mock-infection with PBS. Serial sections of the lungs were stained with HE, TUNEL to identify apoptotic and dying cells, and anti-O2 antibody to identify MT78 and IMT5155.

**Figure 1:**
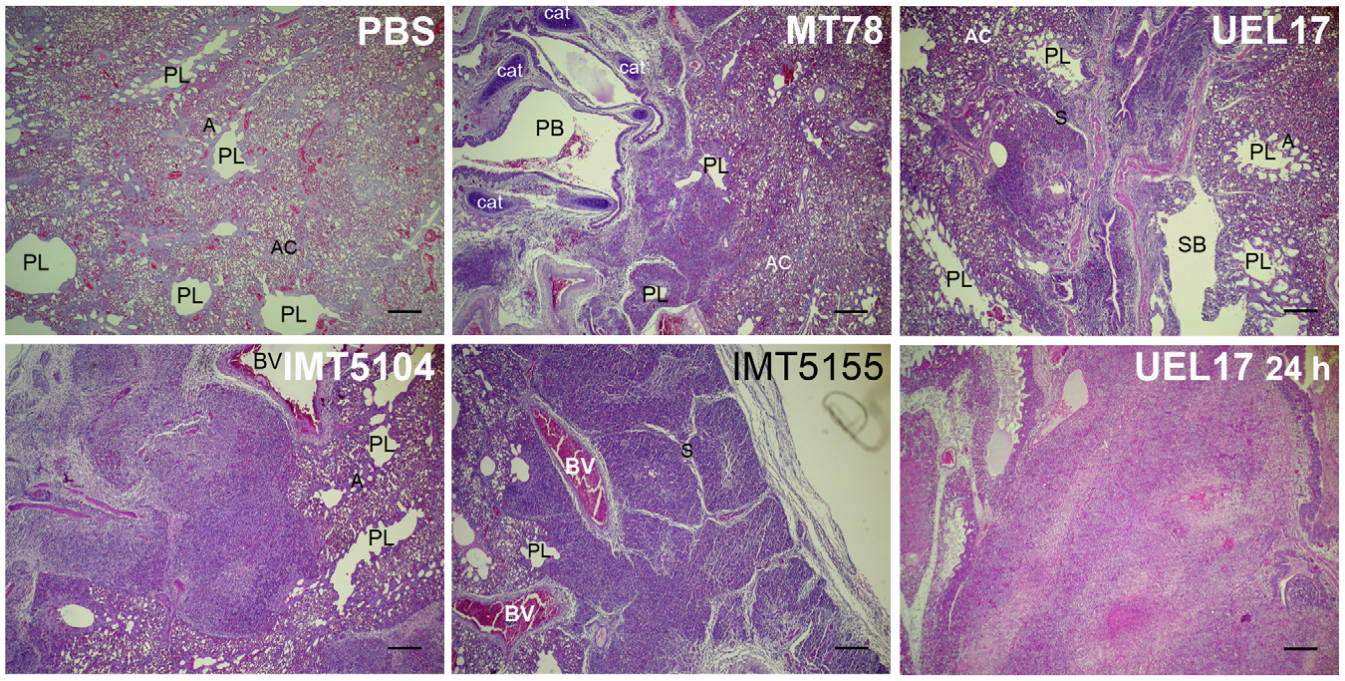
Lungs from chickens infected intratracheally with PBS (*upper left*) or 10^9^ CFU of APEC MT78 (*upper centre*), UEL17 (*upper right*), IMT5155 (*lower centre*), and A_fecal_ IMT5104 (*lower left*). Lungs were fixed at 12 h p.i.. Lungs from UEL17-infected chicken fixed at 24 h p.i. are also shown (*lower right*). Sections were stained with HE and examined under a light microscope. The lungs of PBS-inoculated chickens had intact parabronchi, and open and aerated atria and air capillaries. The lungs of *E. coli*-infected chickens had inflamed areas next to intact areas. Scale bars 200 µm. PB, primary bronchus; SB, secondary bronchus; PL, parabronchial lumen; A, atria; AC, air capillary; S, interparabronchial septa; BV, blood vessel; Cat, cartilaginous plates supporting primary bronchus.

In the lungs of PBS-inoculated chickens, the parabronchial structures were intact at all time points, with the parabronchi lumens and atria open and aerated (Fig. 1). Red blood cells were abundant, indicating that air-blood exchange was occurring efficiently. In the lungs from the *E. coli*-infected chickens, in contrast, we observed highly inflamed and congested areas (Fig. 1), characterized by parabronchi without air in the capillary areas; the central lumens were often, but not always, clogged with exudate. In contrast to lungs from the mock-infected chickens, these affected regions contained fewer erythrocytes, but a higher number of lymphocytes, macrophages and heterophils. With MT78, IMT5155 and IMT5104 (A_fecal_), lesions were already evident at 12 h p.i., and did not worsen as infection progressed (at 18 h and 24 h). Infection with UEL17 caused less severe inflammation of the lung at 12 h p.i., with more intense lesions observed at 24 h p.i. (Fig. 1).

**Figure 2:**
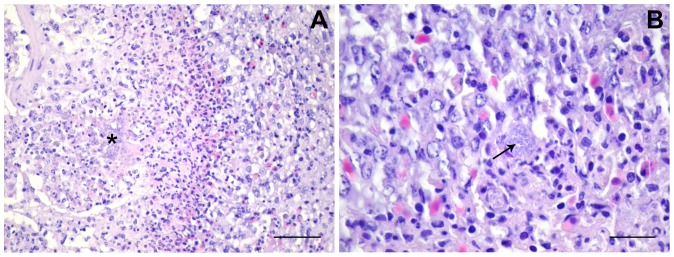
Necrotic foci in *E. coli*-infected lungs. (A) UEL17-infected lungs. The boundary/edge of a necrotic focus containing extracellular bacteria (*). Scale bar 50 µm. (B) IMT5155-infected lungs. Arrow shows a macrophage with intracellular bacteria on the edge of a necrotic focus. Scale bar 20 µm.

**Figure 3:**
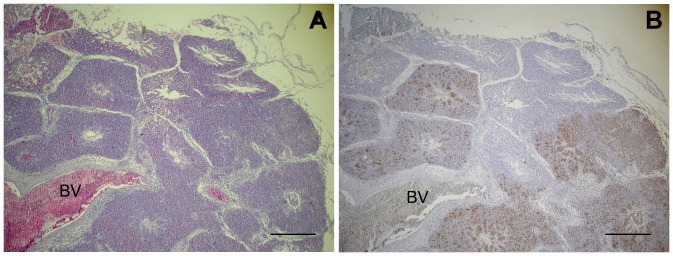
Lungs from chickens infected intratracheally with IMT5155 (10^9^ CFU). Lungs were fixed 24 h p.i.; sections were stained with (A) HE or (B) anti-O2 antibody and counter-stained with hematoxylin, and examined with a light microscope. Inflamed parabronchi coincide with the presence of bacteria, labeled with anti-O2. Scale bars 400 µm. BV, blood vessel.

With all the strains we observed groups of extracellular bacteria, and also macrophages with intracellular bacteria. At 18 h and/or 24 h p.i., all strains produced necrotic foci containing groups of extracellular bacteria and infected macrophages, surrounded by further potentially infected macrophages (Fig. 2). In MT78-infected lungs, necrotic foci could be seen already at 12 h p.i. None of the strains produced necrotic foci with evidence of fibrin deposition. Even when severe inflammation occurred, congested areas were neighbored by unaffected, intact regions in which the parabronchial structures looked intact (Fig. 1 and Fig. 3). The existence of clear, unaffected areas was consistent with the fact that the birds were still alive and breathing.

Labeling sections of MT78 and IMT5155-infected lungs with anti-O2 antibody revealed that the inflamed and congested areas coincided with the presence of bacteria (Fig. 3), which suggests that the inflammation in a given area of lung is a direct consequence of localized infection.

**Figure 4:**
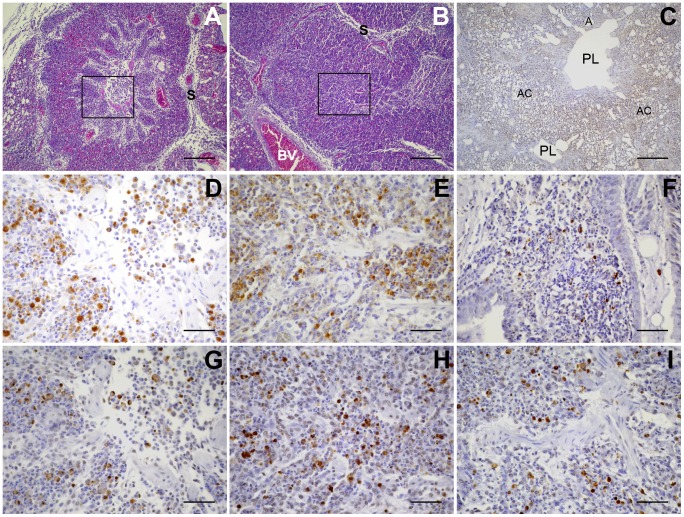
TUNEL-positive cells in the lungs of APEC-infected chickens. Lungs were fixed at 12 h p.i. (A) Clogged parabronchus of MT78-infected chicken, stained with HE; the boxed region is amplified and was stained with anti-O2 antibody in (D), and by TUNEL in (G). (B) Clogged parabronchus of IMT5155-infected chicken, stained with HE; the boxed region is amplified and was stained with anti-O2 antibody in (E), and by TUNEL in (H). (C) Lung section from PBS-inoculated chicken, showing intact parabronchi, open and aerated atria and air capillaries, and absence of TUNEL-positive cells. (F) Parabronchus of UEL17-infected chicken, stained by TUNEL. (I) Parabronchus of IMT5104-infected chicken, stained by TUNEL. All *E. coli*-infected chickens had TUNEL-positive cells in inflamed lung areas. Scale bars, A, B and C, 200 µm; D–I, 50 µm. PL, parabronchial lumen; A, atria; AC, air capillary; S, interparabronchial septa; BV, blood vessel.

Histological sections were also labeled by TUNEL, which identifies the nuclear DNA fragmentation in apoptotic cells. In lungs from PBS mock-infected chickens, virtually no TUNEL-positive cells could be found (Fig. 4). In contrast, infections with all four strains of *E. coli* produced TUNEL-positive cells in the lungs. These cells were restricted to the inflamed areas, which were also positive for bacteria in MT78- and IMT5155-infected lungs. TUNEL-positive cells were evident already at 12 h p.i., although necrotic foci were observed only from 18 h p.i. onwards with IMT5155 and IMT5104, and at 24 h p.i. with UEL17. In agreement with the less severe inflammation of the lungs, infection with UEL17 produced less TUNEL-positive cells at 12 and 18 h p.i. than any of the other strains.

Despite these minor differences, overall the histopathological changes in the lungs caused by the septicemic MT78 and IMT5155 strains and the non-septicemic UEL17 or non-pathogenic IMT5104 strains were very similar.

### Infection of HD11 chicken macrophages with the APEC and IMT5104 strains

**Figure 5:**
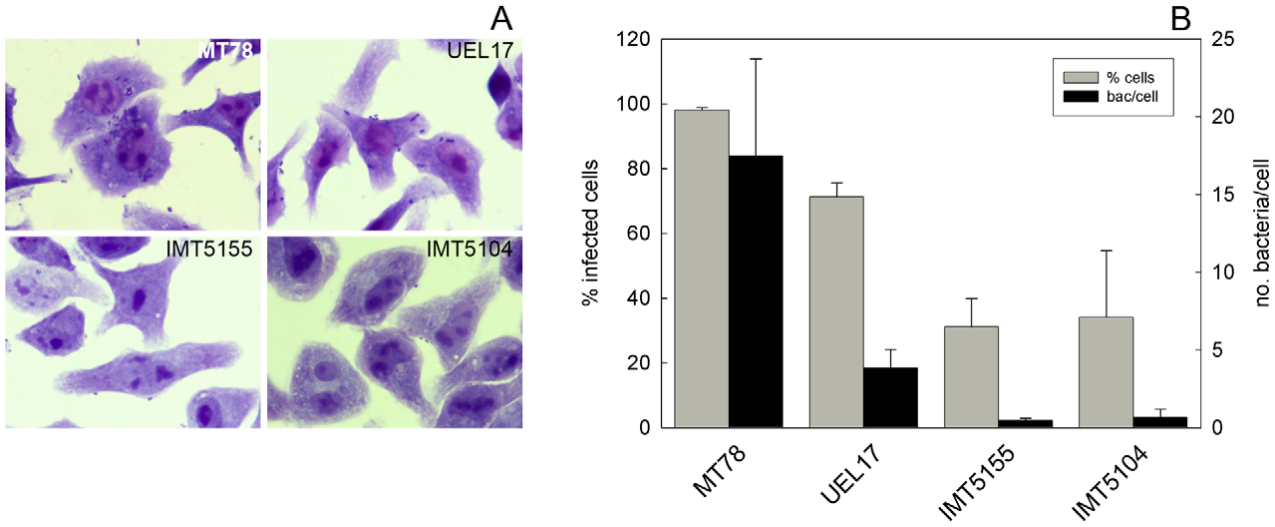
Association of APEC with HD11 chicken macrophages. Cells were infected at a MOI of 150 CFU/cell as described in Section 2.4. (A) At 6 h p.i., the association of bacteria with macrophages was visualized by Giemsa staining. Magnification is 1000×. (B) Graph shows percentage of infected cells (grey bars) and number of associated bacteria per cell (black bars) for each strain. Data are mean ± standard deviation of three experiments performed in triplicate. 100–200 cells were counted in each sample.

In an attempt to elucidate which cells in the lung were TUNEL-positive following infection with avian *E. coli* strains, we infected monolayer cultures of HD11 chicken macrophages with MT78, UEL17, IMT5155 and IMT5104 (A_fecal_) and analyzed the numbers of bacteria associated with the cells, and caspase-3/7 activation in cell extracts. With MT78 and UEL17, many bacteria became associated with the HD11 cells, while with IMT5155 and IMT5104 strains, only a few became associated with the cells (Fig. 5).

**Figure 6:**
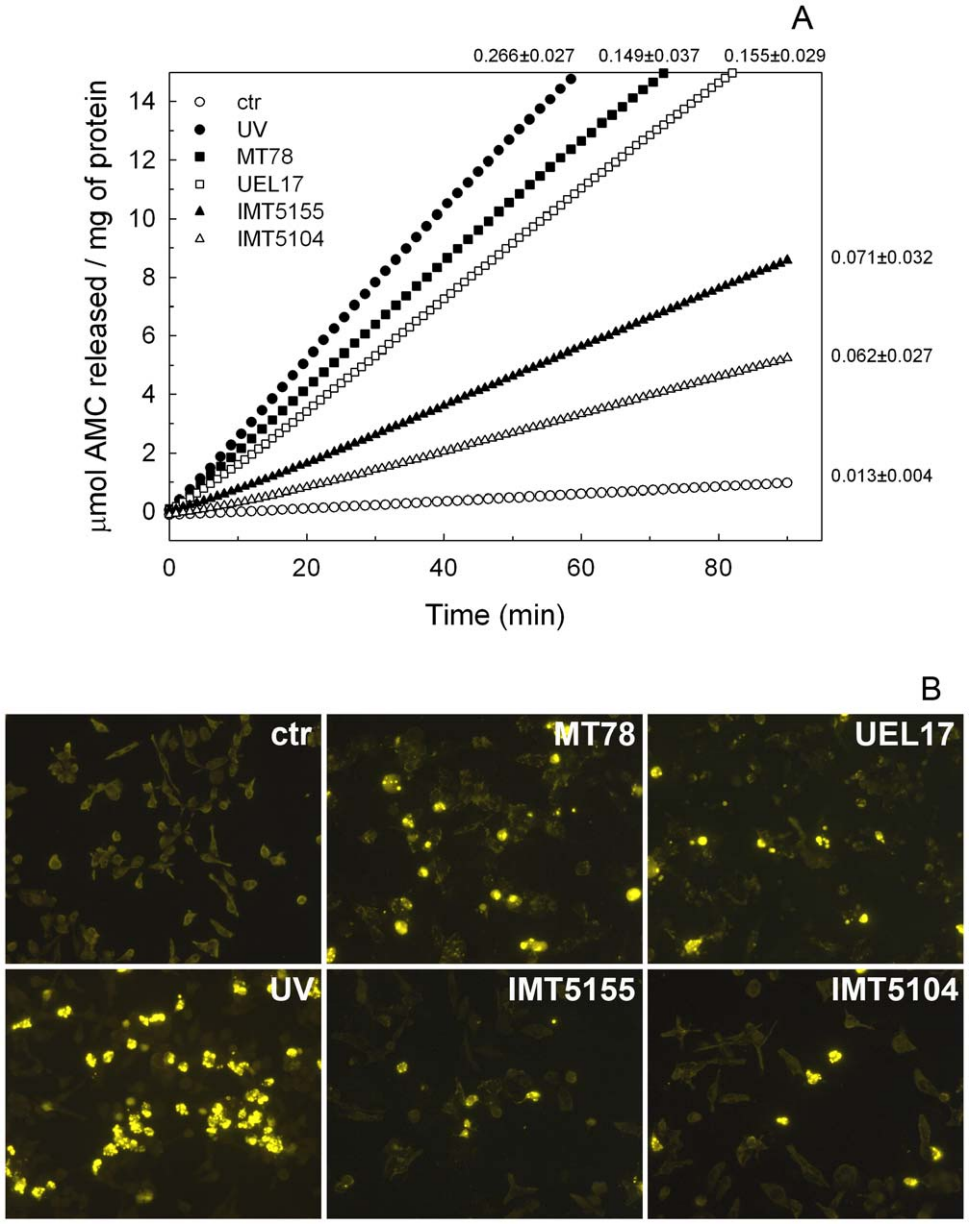
(A) Hydrolysis of caspase 3/7 substrate by cell extracts from *E. coli*-infected HD11 macrophages. Macrophages were infected with MT78 (closed squares), UEL17 (open squares), IMT5155 (closed triangles) or IMT5104 (open triangles) at MOI of 150 CFU per cell. At 6 h p.i., cell extracts were prepared and analyzed using the caspase 3/7 substrate as described in Section 2.5. Open circles, uninfected cells; closed circles, cells irradiated with 0.02 J UV (positive control). Data are representative of at least four experiments performed in duplicate; the mean ± standard deviation of *V*_max_ for each curve is indicated on the right of the curve. (B) TUNEL analysis of HD11 cells. Macrophages were treated as in (A), and after 8 h, samples were fixed with formaldehyde (3.7% in PBS) and analyzed for TUNEL-positive cells (yellow).

To verify if the bacterium-HD11 association results in activation of the apoptotic cascade, cell extracts were prepared 6 h p.i. and tested for caspase activity using a synthetic substrate. All the strains induced caspase 3/7 activation in the infected macrophages, although activation was stronger in the cells infected with MT78 and UEL17 than in those infected with IMT5155 and IMT5104 (Fig. 6A). Caspase 3/7 activation led to DNA degradation, as observed by TUNEL (Fig. 6B). The higher level of caspase 3/7 activation in MT78- and UEL17-infected HD11 macrophages was probably due to the greater number of bacteria associated with macrophages.

**Figure 7:**
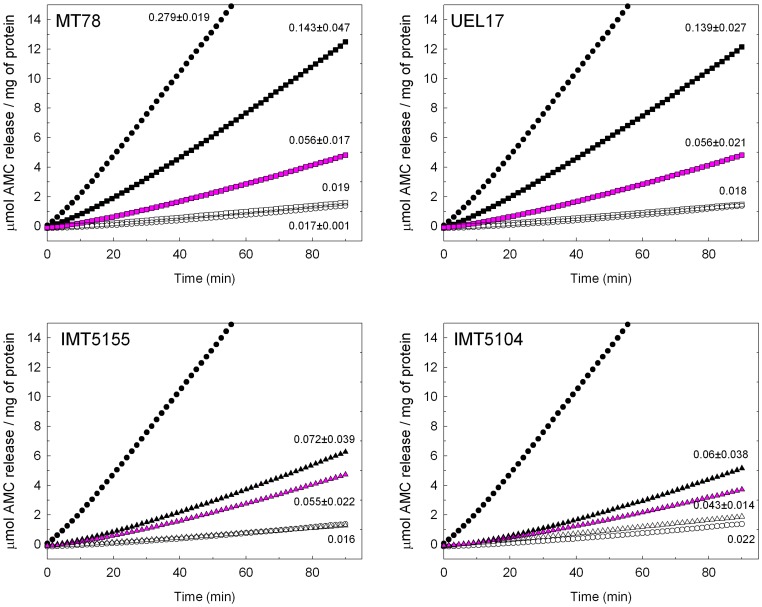
The effect of bacterial inactivation on APEC-induced HD11 macrophage apoptosis. Macrophages were infected with viable bacteria (closed symbols), UV-inactivated bacteria (pink symbols) or UV-inactivated bacteria in the presence of 5 µg/mL polymixin B (open symbols) and caspase activation was analyzed as described in the legend to Fig. 5. Open circles, uninfected cells; closed circles, cells irradiated with 0.02 J UV (positive control). For viable bacteria and UV-inactivated bacteria data are from two experiments performed in duplicate; the mean ± standard deviation of *V*_max_ for each curve is indicated on the right of the curve. For UV-inactivated bacteria in the presence of polymixin B, data are from one experiment.

We next investigated if the bacteria had to be viable to activate caspase 3/7 in infected HD11 macrophages. Caspase 3/7 activation was reduced when the macrophages were infected with UV-inactivated bacteria, and the reduction was more pronounced for strains MT78 and UEL17, so that there was a similar level of caspase 3/7 activation with all four strains (Fig. 7). Since bacterial lipopolysaccharide (LPS) remains intact after UV inactivation, the caspase activation induced by the UV-inactivated bacteria might have been due to the bacterial LPS. To test this possibility, we treated inactivated bacteria with polymixin B, which inhibits the action of LPS; this abolished caspase 3/7 activation, and similar results were obtained when bacterial inactivation was achieved by heating (not shown). These findings suggest that (1) bacterial viability is essential for the high levels of caspase 3/7 activation observed in MT78 and UEL17-infected macrophages, and (2) the caspase 3/7 activation observed with inactivated bacteria is caused by bacterial LPS.

Table 4 summarizes the main properties of the four *E. coli* strains *in vivo* and in monolayers of HD11 macrophages.

**Table 4:** Numbers of virulence-associated genes and properties of the avian *E. coli* strains *in vivo* and in monolayers of HD11 macrophages.

*Properties*	*Strains*			
	MT78	IMT5155	UEL17	IMT5104
Number of virulence-associated genes*	23	25	18	5
Mean lesion scores in all organs	6.6	7.5^a^	5.2	n.d.
Systemic infection	yes	yes	no	no
Extent of lung colonization (CFU)	10^8^	10^8^ a	10^6^	10^6^ b
Focal infection and inflammation in the lung	+++	+++	++	+++
TUNEL-positive cells in the lung	+++	+++	++	+++
Association with HD11 macrophages	+++	+	+++	+
Caspase 3/7 activation in HD11 macrophages induced by viable bacteria	+++	+	+++	+
Caspase 3/7 activation in HD11 macrophages induced by UV-inactivated bacteria	+	+	+	+

* total no. of genes tested: 46 [14]; n.d. = not determined; − = no; + = low; ++ = medium; +++ = high.

a [2].

b [5].

## Discussion

We used a systemic infection model in 5-week-old White Leghorn chickens to compare the histopathological changes in lungs caused by infection with three APEC strains and one A_fecal_ strain. Previous work had already shown that intratracheal inoculation with 10^9^ CFU of IMT5155 caused systemic infection [2], whereas IMT5104 (A_fecal_) remained restricted to the lungs [5]. Here we observed that MT78 caused a systemic infection in 5-week-old chickens, while UEL17 was restricted to the lungs. Moreover, the levels of lung colonization with MT78 were comparable to those with IMT5155 (∼10^8^ CFU/g of lung tissue at 24 h p.i), and the levels for UEL17 were similar to those with IMT5104 (∼10^6^ CFU/g of lung tissue at 24 h p.i.). APEC UEL17 was isolated from a chicken with generalized infection in 1990 and had been considered a highly virulent strain based on virulence tests in 1-day-old chickens [18]. It was thus rather surprising that it did not prove virulent in 5-week-old chickens, although it is possible that virulence in 1-day-old chickens does not necessarily reflect virulence in older chickens. A more probable explanation is that UEL17 has lost some virulence factors during storage over the years. Even though, UEL17 still harbors several genes associated with APEC virulence: apart from the absence of genes for invasins *ibeA* and *gimB*, absence of K1-capsule encoding gene and the presence of *pic*, the virulence-associated genotype of UEL17 is virtually the same as those of MT78 and IMT5155 (Table 1), nonetheless UEL17 behaved as the non-pathogenic IMT5104 in 5-week-old chickens.

When we examined lung sections from infected chickens, we observed that despite the fact that lung colonization by UEL17 and IMT5104 was two orders of magnitude less than by MT78 and IMT5155, all four strains caused the same extent of inflammation. Inflammation was restricted to defined areas in the lung in which air capillaries were collapsed and a large number of leukocytes were present. The histopathological lung changes observed in this study were essentially the same as those described for *E. coli* strain 506 (O78:K80) [15].

Anti-O2 antibody labeling of MT78 and IMT5155 revealed that bacteria were present only in the inflamed areas of the lung. It was remarkable how inflammation remained restricted to the infected areas. Although we failed to label UEL17 and IMT5104 using a commercial anti-*E. coli* antibody, we would predict that the localized inflammation observed in lungs infected with these two strains was also a result of localized bacterial infection. While the inflammation due to infection with UEL17 was more severe at 24 h p.i., the inflammation due to infection with APEC MT78 and IMT5155 and A_fecal_ IMT5104 was slightly more severe at 12 h p.i., and with all strains purulent inflammation developed into necrotic foci at later time points, suggesting that the birds would succeed in resolving the lung infection by isolating the infected regions. Infection with all strains produced TUNEL-positive dying cells in the lungs at all time points (again only in the inflamed areas). Thus, colonization of the chicken lung by an avian *E. coli* strain is accompanied by apoptosis, irrespective of its ability to infect internal organs. At later time points, TUNEL-positive cells were also found in the necrotic foci, as expected.

It has been found that 5-week-old White Leghorn chickens infected intratracheally with APEC at dose below 10^6^ CFU develop only localized lung infections, whereas if the dose is increased to 10^9^ CFU, lung infection spreads to the bloodstream and becomes systemic [2]. We chose a systemic infection model because we wanted to see if a systemic APEC could be distinguished from a non-systemic strain in terms of the histopathological changes caused in avian lungs. Overall, we could not distinguish an APEC from an A_fecal_ *E. coli* by looking at the histopathology of the infected lungs. One possible explanation is that the systemic infection model is not adequate for investigating lung histopathology because any strain would cause the observed symptoms when inoculated intratracheally at a dose of 10^9^ CFU. If, however, this model does reproduce what happens in the field, then pathogenic and non-pathogenic *E. coli* strains do indeed cause similar histopathological changes in the avian lung. It is interesting that Dwars *et al.* observed virtually no differences in the histopathology of lungs infected with *E. coli* or with *E. coli* plus infectious bronchitis virus [15].

We next wanted to investigate which cells were dying in the infected areas of the avian lung. The blood-gas barrier of the avian lung is essentially formed by endothelial cells (67%), extracellular matrix (21%) and squamous epithelial cells (12%) [25]. In an infection, phagocytes, such as heterophils and macrophages present in bronchus-associated lymphoid tissue (BALT) nodules and interstitia [26], are rapidly recruited to the sites of infection. Thus endothelial and epithelial cells, heterophils, and macrophages are potential candidates for the observed dying cells. Of those, the most likely candidates to be dying at the infection site are the cells involved in defense. In previous work, none of the avian strains studied here were cytotoxic or induced caspase 3/7 activation in chicken fibroblasts [19], which, like endothelial and epithelial cells, are non-phagocytic. On the other hand, it is known that neutrophils, the mammalian counterparts of avian heterophils, undergo apoptosis following ingestion of microorganisms during infections [27]. Heterophils are the first line of defense and die at the infection site, as evidenced by purulent inflammation (Fig. 1). Previously, purulent necrosis of parabronchi has been found in lungs infected with *E. coli* strain 506 (O78:K80) [15], and degenerating heterophils were observed by transmission electron microscopy in the lung air capillaries of chickens inoculated with APEC [28]. Therefore, some of the TUNEL-positive cells are very likely dying heterophils.

Since we had previously observed apoptosis of murine macrophages following UEL17 infection [29], we wondered whether there could also be dying macrophages in the infected lungs. To test this idea we used monolayer cultures of HD11 cells to investigate whether infection with APEC would induce apoptosis in avian macrophages *in vitro*. All four avian *E. coli* strains induced caspase 3/7 activation in these monolayers, suggesting that the dying cells observed in avian lungs *in situ* could include macrophages. *In vitro*, MT78 and UEL17 induced higher levels of caspase 3/7 activation in infected HD11 macrophages than IMT5155 and IMT5104, and this likely resulted from the higher numbers of MT78 and UEL17 associated with HD11 (Fig. 5). This is consistent with previous observations that MT78 (O2) associates at higher levels with avian heterophils and macrophages than χ7122 (O78) and TK3 (O1) strains [30]. Interestingly, MT78 and IMT5155 are both serogroup O2, ST complex 95 and share several virulence-associated genes [19], yet associated with quite different efficiencies with the macrophages (Fig. 5).

In addition to the extent of bacterial association with macrophages, bacterial viability was a factor in inducing the higher levels of caspase 3/7 activation of MT78- and UEL17-infected macrophages; when they were inactivated all four strains induced similar levels of caspase 3/7 activation (Fig. 7). The caspase activation induced by inactivated bacteria is probably due to LPS, a hypothesis confirmed by the complete inhibition of caspase activation in the presence of the antagonist of LPS, polymixin B. Because viable MT78 and UEL17 induced higher levels of caspase activation in HD11 macrophages than IMT5155 and IMT5104, but only MT78 and IMT5155 caused systemic infection in 5-week-old chickens, we conclude that the induction of HD11 macrophage apoptosis does not reflect a strain's virulence.

Our *in vitro* results suggest that MT78 and UEL17 induce apoptosis of a higher number of macrophages than IMT5155 and IMT5104, yet the numbers of TUNEL-positive cells *in situ* in lungs infected with MT78, IMT5155 and IMT5104 strains were similar. One explanation is that *in situ* the large numbers of TUNEL-positive heterophils mask any differences in the number of TUNEL-positive macrophages. Alternatively, macrophage death *in vivo* may be determined solely/mainly by bacterial LPS. Unfortunately, labeling with the macrophage antibody KUL01 is only possible in cryosections [31], not in paraffin-embedded tissue, so it was not possible to double-label the sections with TUNEL and KUL01.

To conclude, intratracheal infection of chickens with four avian *E. coli* strains produced localized lung infections with purulent inflammation evident by 12 h. p.i. There were no differences in lung histopathology that could distinguish pathogenic from a non-pathogenic avian *E. coli* strains. Moreover, our *in vitro* results suggest that the dying cells in the lung may be macrophages as well as heterophils; however the ability of a strain to induce higher levels of apoptosis in HD11 macrophages was not correlated with its virulence *in vivo*.
